# Association between Butter, Margarine, and Olive Oil Intake and Asthma Symptoms among School Children: Result from a Large-Scale Cross-Sectional Study

**DOI:** 10.1155/2023/2884630

**Published:** 2023-10-18

**Authors:** Arezoo Sadat Emrani, Bahareh Sasanfar, Zahra Nafei, Nasrin Behniafard, Amin Salehi-Abargouei

**Affiliations:** ^1^Research Center for Food Hygiene and Safety, School of Public Health, Shahid Sadoughi University of Medical Sciences, Yazd, Iran; ^2^Yazd Cardiovascular Research Center, Non-communicable Diseases Research Institute, Shahid Sadoughi University of Medical Sciences, Yazd, Iran; ^3^Department of Nutrition, School of Public Health, Shahid Sadoughi University of Medical Sciences, Yazd, Iran; ^4^Cancer Research Center, Cancer Institute of Iran, Tehran University of Medical Sciences, Tehran, Iran; ^5^Children Growth Disorder Research Center, Shahid Sadoughi University of Medical Sciences, Yazd, Iran; ^6^Department of Allergy and Clinical Immunology, Shahid Sadoughi University of Medical Sciences, Yazd, Iran

## Abstract

**Background:**

There are conflicting results about the association between dietary fat intake and asthma symptoms. Since few studies in the Middle East have been explored the relation between dietary fat consumption and risk of asthma, the present study was conducted to investigate the association between the consumption of butter, margarine, and olive oil and asthma risk in school children living in central Iran.

**Method:**

In this cross-sectional study, out of 10,240 participants, asthma and its symptoms and dietary intake of butter, margarine, and olive oil of 7,667 children and adolescents were assessed using a validated International Study of Asthma and Allergies in Childhood (ISAAC) questionnaire. The relationship between fat subtypes and asthma was assessed using logistic regression.

**Results:**

The prevalence of asthma confirmed by a doctor in the study population was 4.22%. An inverse association was found between butter and margarine consumption once or twice a week and odds of current asthma and wheezing in the past 12 months (OR = 0.52, 95% CI: 0.28–0.96; OR = 0.7, 95% CI: 0.55–0.88, respectively); however, those with higher consumption did not have a higher chance for developing wheezing or asthma.

**Conclusion:**

We found that margarine and butter intake one or two times a week might have an inverse association with asthma and its symptoms among children. Prospective cohort studies are recommended to confirm these findings.

## 1. Introduction

Asthma, inflammation, and narrowing of the airways are characterized by symptoms such as shortness of breath, cough, and wheezing and are one of the most common chronic respiratory diseases among children. This respiratory disease has affected about 262 million children and adults in 2019 and caused 461,000 deaths across the world [1]. A lower prevalence of asthma was reported in the Middle East than in developed countries [2]. The prevalence reported in the countries of this region is 14.3% in Saudi Arabia [3], 17.8% in Turkey [4], and 8.9% in Iran [5].

Body mass index, smoking, race, urbanization [6], and genetics [7] are some of the risk factors for asthma. Diet also plays an important role and many studies have shown that patients may benefit from a diet that is rich in fresh vegetables and fruits [8]. In contrast, an obesogenic diet that includes a high intake of processed foods and saturated fats and a low intake of fruits, vegetables, and whole grains may lead to inflammation and the development of chronic diseases such as asthma [9]. Also, one study indicated that a diet high in fat, sugar, and salt is associated with a higher prevalence of severe asthma [10].

Today, the Western diet is becoming a common dietary pattern in the Middle East, and the adherence to traditional dietary patterns has decreased [11]. Western dietary pattern is characterized by a high intake of sugar, refined grains, meat, and fats and a low intake of vegetables, fruits, and whole grains [11]. The Iranian dietary pattern is also rich in refined grains such as rice and bread (especially lavash bread), fried chicken, processed foods, and carbonated beverages [12]. Also, one study using detailed in-home assessments of dietary intake demonstrated that Iranian families consume almost twice as much trans fatty acids (especially from hydrogenated oils) as Americans [13].

According to studies, some fatty acids have anti-inflammatory and others have proinflammatory properties, and, through this, they can affect respiratory diseases [14]. However, there are conflicting results regarding the effect of different types of dietary fat on asthma. Some studies have shown that fish and fish oil intake may prevent the development of asthma in children [15]. A population-based cross-sectional study with a sample size of 1,166 adolescents aged 13–17 years found that consumption of foods rich in protein and fat from animal sources may increase the risk of asthma in adolescents [16]. However, in a cohort study, no association was found between fatty acid intake and asthma [17]. Rodríguez-Rodríguez et al. [18] showed that the intake of saturated fatty acids (SFAs) such as myristic and palmitic acids (and butter as the main source of these two fatty acids) is associated with a higher risk of current asthma in children. Also, another study found that SFA intake was associated with an increased risk of asthma, while consumption of monounsaturated fatty acids was inversely related to the disease [19]. To the best of our knowledge, there is one study with a sample size of 1,000 children that has explored the relationship between olive oil intake and asthma in the Middle East [20]. This study revealed that daily consumption of olive oil is associated with higher odds of current asthma; however, adherence to the Mediterranean diet was associated with lower odds of current asthma [20].

The aim of this study is to evaluate the relationship between dietary margarine, butter, and olive oil intake among 6–7- and 13–14-year-old school children in Yazd city, central Iran.

## 2. Subjects and Methods

### 2.1. Participants

This cross-sectional study, which was conducted in early 2020 in Yazd city, was conducted as part of the Global Asthma Network (GAN). The GAN is a cross-sectional, multicountry, multicenter, epidemiological research that follows and expands the methodology used in the International Study of Asthma and Allergies in Childhood (ISAAC) Phase III [21]. According to the GAN recommendation, a sample size of at least 3,000 participants is required to accurately estimate the prevalence of asthma [22]. In the present study, students from 36 elementary and 48 high schools (state and private) were randomly selected. Samples were taken from two educational districts using a cluster sampling design and non-Iranian students were excluded. Due to the school closures during the COVID-19 pandemic, all participants aged 13–14 years and parents of participants aged 6–7 years were asked to complete online electronic questionnaires on asthma and its symptoms and risk factors that were placed in the virtual education groups of schools. However, the data of several participants aged 6–8 years were collected through a paper questionnaire before the COVID-19 quarantine. Out of 7,214 children and 3,026 adolescents, 5,141 and 2,526 completed the questionnaire (response rate: 71.3% and 83.5%, respectively). Then, demographic data that seemed to be unacceptable were checked through a telephone call and were corrected if necessary.

The ethics committee of Shahid Sadoughi University (SSU) of Medical Sciences, Yazd, Iran (IR.SSU.REC.1398.244) ethically approved the GAN study on Iranian children. The present study was also ethically approved by the ethics committee (ethics approval code: IR.SSU.SPH.REC.1400.135). The permission to conduct the study in schools was obtained from the Yazd Education administration. Informed consent was obtained from all participants or the parents of the participants.

### 2.2. Asthma and Its Symptoms Confirmation

The GAN questionnaire, which was used in our study and derived from the ISAAC questionnaire [23], includes questions about the symptoms of allergic diseases and their related risk factors. In this study, there were some questions about asthma confirmation, including “use of asthma medication” and “asthma confirmed by a doctor”, as well as its symptoms, including wheezing in the past 12 months, eczema, and rhinitis. As it is mentioned in the protocol of this study, current asthma was defined as a history of confirmed asthma by a doctor and having had wheezing and/or use of asthma medication in the past 12 months. After translating the questionnaire into Persian, a study with 100 selected subjects was conducted to confirm the reliability of the translated version using Cronbach's alpha. The alpha coefficient for asthma symptoms was estimated to be 0.862, which exhibits appropriate internal consistency. Finally, the questionnaire was translated back into English and sent to the GAN principals for approval.

### 2.3. Assessment of Dietary Intakes

In the current study, usually dietary intakes of children's food groups in the last 12 months were assessed using the GAN questionnaire that included multiple choice questions [24]. In the answer section, three options showed the frequency of food consumption (never or occasionally/once or twice a week/most or all days of the week). Olive oil, margarine, and butter consumption were used to assess their relationship with asthma and its symptoms.

### 2.4. Assessment of Other Variables

Data on participants' demographic characteristics, including their height, weight, and ethnicity (Kord, Turk, Persian, Lor, Arab, Balooch), and other variables such as watching TV and computer use (2–4/5–8/9–14 hr a day) were obtained using a self-reported online GAN questionnaire. The body mass index (BMI) was also calculated using the following formula: weight (kg)/height squared (m^2^).

### 2.5. Statistical Methods

All analyses were performed using STATA software version 14 (STATA Corp., Lakeway Drive, USA). *χ*^2^ test and independent sample *t*-test were used to compare ordinal qualitative variables and continuous variables in children with or without asthma confirmed by a doctor, respectively. Logistic regression was used to assess the association between dietary butter, margarine, and olive oil intake and asthma confirmed by a doctor, current asthma, and wheezing in the past 12 months in crude models. These analyses were also done in multivariable-controlled models to adjust possible confounder variables, including participants' age and sex, watching TV and computer use, and BMI. The reference category was the lowest frequency of dietary intake (never or only occasionally). *P*-value < 0.05 was considered as statistically significant.

## 3. Results

The characteristics of the 7,667 participants who filled out all questionnaires are shown in Table 1. The prevalence of asthma confirmed by a doctor was 4.22% in this population. Children with asthma confirmed by a doctor had significantly more wheezing at any time in the past, wheezing in the past 12 months, and use of asthma medication than children without asthma confirmed by a doctor (*P*  < 0.001).

The association between dietary butter, margarine, and olive oil intake and asthma confirmed by a doctor is presented in Table 2. An increasing trend but no significant association was found between margarine, butter, and olive oil intake with asthma confirmed by a doctor (Table 2). There was also no significant association between odds of current asthma and butter intake in the crude model. However, after adjusting for age and sex, participants in the second tertile had 48% lower odds of current asthma compared to those in the first tertile (OR = 0.52, 95% CI: 0.28–0.96). This association remained significant after adjusting for further confounders (OR = 0.52, 95% CI: 0.28–0.96). A significant inverse trend was also found in the association between butter intake and current asthma (*P*_trend_ = 0.05) (Table 3).

As shown in Table 4, the association between dietary butter, margarine, and olive oil intake with wheezing in the past 12 months was reported. Participants who consumed margarine once or twice per week had lower odds of wheezing in the past 12 months compared to those with the lowest intake (OR = 0.7, 95% CI: 0.55–0.88). A significant trend was also observed (*P*_trend_ = 0.02).

## 4. Discussion

Our findings showed an inverse association exists between moderate butter consumption once or twice a week and odds of current asthma. Also, the same relation was observed between margarine intake and wheezing in the past 12 months. However, in higher consumption, these associations were not significant.

In our study, children consumed butter once or twice a week had a lower odds of current asthma than those never or occasionally consumed this food. Previous studies have also investigated the association between butter intake and the odds of asthma. In the Prevention and Incidence of Asthma and Mite Allergy (PIAMA) birth cohort study, Wijga et al. [25] examined the role of diet in the development of asthma in preschool children. Their results indicated that the incidence of asthma in children who consumed butter daily was lower than in those who did not. Also, similar results were found in a cross-sectional study in which butter consumption was inversely associated with doctor-diagnosed asthma [26]. Another study examined the relationship between dietary fatty acids composition during pregnancy and the incidence of asthma in infants, and illustrated that maternal intake of SFAs (especially palmitic acid, of which butter is the main source) may decrease the risk of asthma in the offspring [27]. One possible explanation for this result is that consuming more unsaturated than saturated fats may be associated with a higher incidence of asthma [28]. There is also evidence that reducing the intake of omega-3 and saturated fatty acids from butter and increasing the intake of omega-6 fatty acids leads to an increase in the ratio of omega-6 to omega-3 intake. This increased ratio results in increasing the production of prostaglandin E2 and related processes, which increase asthma and atopic disease symptoms [29]. In contrast, a cross-sectional study conducted on Spanish school children found a positive relation between butter consumption and current asthma [18]. Another cross-sectional study on female university students in Japan also suggested that those who consume more butter experienced wheezing more [30]. Moreover, the results of a cross-sectional study using the ISAAC questionnaire in Columbia showed that in the multivariate model, a weekly intake of butter is not related to asthma symptoms [31]. The differences between dietary patterns of the mentioned studies' population within our study could be an explanation for the inconsistent findings.

There was also an inverse association between margarine consumption one or two times a week and wheezing in the past 12 months. Similarly, Kim et al. [32] examined the relationship between diet and the school environment with asthma and allergies and showed that consuming margarine is related to lower likelihood of wheezing. However, the results of some studies were inconsistent with our results. Phase III of the ISAAC which was a cross-sectional study showed that margarine consumption by children might increase the risk of wheezing [33]. Also, the German National Health Survey in 1998 showed that higher consumption of margarine is associated with current asthma [34]. Margarine is rich in polyunsaturated fatty acids (PUFAs), and it is hypothesized that a high intake of omega-6 PUFAs may be associated with chronic lung disease [35]. Also, it is mentioned that high margarine intake is associated with low butter intake, and it has been suggested that higher margarine consumption may be related to a higher incidence of asthma by lower butter intake [32]. This conflict between our results and other studies may be due to either different dietary pattern intake of Iranians, which contains a higher intake of carbohydrates [12] and saturated fat [36] or different compositions of margarine produced in Iran compared to other countries [37]. Iranian margarine has a high amount of SFA, especially palmitic acid [37], which is related to lower asthma risk as discussed earlier. This may explain the reducing effect of margarine on wheezing in the present study.

In this study, no association was found between olive oil intake and asthma. Previous investigations have led to inconsistent results in this regard. Although some studies have shown the protective effect of olive oil ingestion on the odds of this respiratory disease [32], the results of a cross-sectional study indicated the additive effects of olive oil consumption on the odds of asthma [20].

This present study benefited from a large sample size, which provide more representative of the population. Several subgroup analyses have been conducted. Furthermore, a valid questionnaire was used for collecting our intended data. However, the current study also has some limitations. The questionnaire used in this study could not provide the grams of food intake, which may result in misclassification of participants. Furthermore, only the data of age, gender, BMI, and watching TV and computer use were obtained through this questionnaire, so we were not able to perform any other adjustment. Since the design of this study was cross-sectional, recall and reporting bias might affect the findings. In addition, casual association cannot be inferred because of the cross-sectional design of the study. However, although causal relationships could not be found from our results, this study was able to hypothesize that there is an association between butter and margarine intake and asthma and provide a good background data for further studies.

In conclusion, it seems that moderate weekly intake of butter and margarine might be associated with the likelihood of asthma and its symptoms. However, higher intakes did not lead to a further reduction in the likelihood of the disease. It is necessary to conduct investigations with stronger design such as cohort studies to confirm these findings.

## Figures and Tables

**Table 1:** General characteristics of the subjects according to asthma confirmed by a doctor.^a^

Variables	Asthma confirmed by a doctor		*P*-Value
	Without (*n* = 7343)	With (*n* = 324)	
Sex			
Boys	3,226 (43.93)	188 (58.02)	<0.001
Girls	4,117 (56.07)	136 (41.98)	
Age (years)	10.9 ± 3.37	11.7 ± 2.94	<0.001
BMI (kg/m^2^)	18.9 ± 10.4	19.1 ± 4.18	0.35
Physical activity (watching TV and computer use)			0.38
2–4 hr	3,945 (53.7)	163 (50.3)	
5–8 hr	2,463 (33.5)	103 (34.8)	
9–14 hr	935 (12.7)	48 (14.8)	
Wheezing (any time in the past)			
Yes	6,183 (84.2)	228 (70.3)	<0.001
No	1,160 (15.8)	96 (29.6)	
Wheezing (in the past 12 months)			
Yes	553 (7.5)	56 (17.2)	<0.001
No	6,790 (92.4)	268 (82.7)	
Use of asthma medication			
Yes	134 (1.8)	57 (17.5)	<0.001
No	7,209 (98.1)	267 (82.4)	
Margarine			
Never	5,432 (73.9)	240 (74.0)	0.99
Weekly	1,558 (21.2)	68 (20.9)	
Every day	353 (4.8)	16 (4.9)	
Butter			
Never	3,734 (50.8)	185 (57.1)	0.06
Weekly	2,780 (37.8)	103 (31.7)	
Every day	829 (11.2)	36 (11.1)	
Olive oil			
Never	5,055 (68.8)	219 (67.5)	0.55
Weekly	1,583 (21.5)	68 (20.9)	
Every day	705 (9.6)	37 (11.4)	

Values are mean (SD) or percentages. ^a^*χ*^2^ test for ordinal qualitative variables and *t*-test for continuous variables.

**Table 2:** Association between fat and fats subtypes intake and asthma confirmed by a doctor.

Fat intake	T1	T2	T3	*P*_trend_
	Never or only occasionally	Once or twice per week	Most or all days	
	OR (95% CI)	OR (95% CI)	OR (95% CI)	
Margarine				
No. of with/without asthma	240/5,432	68/1,558	16/353	
Crude	1.00	0.98 (0.75–1.3)	1.02 (0.61–1.72)	0.99
Model 1	1.00	1.07 (0.81–1.41)	1.15 (0.68–1.93)	0.49
Model 2	1.00	1.07 (0.81–1.42)	1.14 (0.68–1.93)	0.48
Model 3	1.00	1.07 (0.81–1.41)	1.14 (0.68–1.93)	0.49
Butter				
No. of with/without asthma	185/3,734	103/2,780	36/829	
Crude	1.00	0.74 (0.58–0.95)	0.87 (0.6–1.26)	0.09
Model 1	1.00	0.74 (0.58–0.95)	0.86 (0.59–1.24)	0.08
Model 2	1.00	0.74 (0.58–0.95)	0.86 (0.59–1.24)	0.08
Model 3	1.00	0.74 (0.58–0.95)	0.86 (0.59–1.24)	0.08
Olive oil				
No. of with/without asthma	219/5,055	68/1,583	37/705	
Crude	1.00	0.99 (0.75–1.30)	1.21 (0.84–1.73)	0.41
Model 1	1.00	0.96 (0.73–1.27)	1.15 (0.80–1.65)	0.59
Model 2	1.00	0.97 (0.73–1.28)	1.16 (0.81–1.66)	0.56
Model 3	1.00	0.97 (0.73–1.28)	1.16 (0.81–1.66)	0.56

Model 1: Adjusted for age and sex. Model 2: Further adjusted for watching TV and computer use. Model 3: Additionally adjustment for BMI.

**Table 3:** Association between fat and fat subtypes intake and current asthma.

Fat intake	T1	T2	T3	*P*_trend_
	Never or only occasionally	Once or twice per week	Most or all days	
	OR (95% CI)	OR (95% CI)	OR (95% CI)	
Margarine				
No. of with/without current asthma	36/4,979	17/1,482	3/329	
Crude	1.00	1.58 (0.88–2.83)	1.26 (038–4.11)	0.2
Model 1	1.00	1.37 (0.76–2.46)	0.97 (0.29–3.22)	0.53
Model 2	1.00	1.37 (0.76–2.46)	0.97 (0.29–3.22)	0.53
Model 3	1.00	1.37 (0.76–2.46)	0.97 (0.29–3.22)	0.53
Butter				
No. of with/without current asthma	36/3,447	15/2,583	5/760	
Crude	1.00	0.55 (0.3–1.01)	0.62 (0.24–1.61)	0.08
Model 1	1.00	0.52 (0.28–0.96)	0.59 (0.23–1.52)	0.05
Model 2	1.00	0.52 (0.28–0.96)	0.59 (0.23–1.52)	0.05
Model 3	1.00	0.52 (0.28–0.96)	0.59 (0.23–1.52)	0.05
Olive oil				
No. of with/without current asthma	41/4,660	8/1,468	7/662	
Crude	1.00	0.61 (0.28–1.32)	1.2 (0.53–2.68)	0.83
Model 1	1.00	0.63 (0.29–1.35)	1.28 (0.57–2.89)	0.95
Model 2	1.00	0.63 (0.29–1.35)	1.28 (0.57–2.89)	0.95
Model 3	1.00	0.63 (0.29–1.35)	1.28 (0.57–2.89)	0.95

Model 1: Adjusted for age and sex. Model 2: Further adjusted for watching TV and computer use. Model 3: Additionally adjustment for BMI.

**Table 4:** Association between fat and fat subtypes intake and wheezing in the past 12 months.

Fat intake	T1	T2	T3	*P*_trend_
	Never or only occasionally	Once or twice per week	Most or all days	
	OR (95% CI)	OR (95% CI)	OR (95% CI)	
Margarine				
No. of with/without wheezing	489/5,183	93/1,533	27/342	
Crude	1.00	0.64 (0.51–0.8)	0.83 (0.55–1.25)	0.002
Model 1	1.00	0.68 (0.54–0.86)	0.9 (0.6–1.36)	0.01
Model 2	1.00	0.7 (0.55–0.88)	0.89 (0.59–1.34)	0.01
Model 3	1.00	0.7 (0.55–0.88)	0.89 (0.59–1.34)	0.02
Butter				
No. of with/without wheezing	323/3,596	212/2,671	74/791	
Crude	1.00	0.88 (0.73–1.05)	1.04 (0.79–1.35)	0.69
Model 1	1.00	0.87 (0.72–1.04)	1.02 (0.78–1.33)	0.58
Model 2	1.00	0.89 (0.74–1.07)	1.04 (0.79–1.36)	0.75
Model 3	1.00	0.89 (0.74–1.07)	1.04 (0.8–1.36)	0.76
Olive oil				
No. of with/without wheezing	436/4,838	123/1,528	50/692	
Crude	1.00	0.89 (0.72–1.09)	0.8 (0.59–1.08)	0.09
Model 1	1.00	0.87 (0.7–1.07)	0.76 (0.56–1.03)	0.04
Model 2	1.00	0.91 (0.73–1.12)	0.79 (0.58–1.08)	0.11
Model 3	1.00	0.9 (0.73–1.12)	0.8 (0.59–1.08)	0.11

Model 1: Adjusted for age and sex. Model 2: Further adjusted for watching TV and computer use. Model 3: Additionally adjustment for BMI.

## Data Availability

The datasets generated and/or analyzed during the current study are available from the corresponding author on reasonable request.
